# Cross-cultural translation and validation of the Spanish version of the patellofemoral pain and osteoarthritis subscale of the KOOS (KOOS-PF)

**DOI:** 10.1186/s13104-021-05619-3

**Published:** 2021-06-02

**Authors:** Juan Pablo Martinez-Cano, Daniel Vernaza-Obando, Julián Chica, Andrés Mauricio Castro

**Affiliations:** 1 Departamento de Ortopedia y Traumatología, Fundación Valle del Lili, Cra 98 # 18 - 49, Consultorio 118, 760032 Cali, Colombia; 2 Universidad Icesi, Calle 18 #, 122-135 Cali, Colombia; 3 Centro de Investigaciones Clínicas, Fundación Valle del Lili, Cra 98 # 18 – 49, 760032 Cali, Colombia

**Keywords:** Patient reported outcome measures, Patellofemoral pain syndrome, Validation study, Translating

## Abstract

**Objective:**

The aim of this study was to translate to Spanish the patellofemoral pain and osteoarthritis subscale of the knee injury and osteoarthritis outcome score (KOOS-PF) and validate this Spanish version of a disease-specific patient-reported outcome measure (PROM) for patellofemoral pain.

**Results:**

The KOOS-PF was translated to Spanish and sixty patients with patellofemoral pain and/or osteoarthritis accepted to complete the questionnaire. 1-week later 58 patients answered the questions again for the test–retest reliability validation and finally 55 patients completed 1-month later for the responsiveness assessment. The Spanish version showed very good internal consistency (Cronbach’s alpha: 0.93) and test–retest reliability (intraclass correlation coefficient: 0.82). Responsiveness was confirmed, showing a strong correlation with the global rating of change (GROC) score (r 0.64). The minimal detectable change was 11.1 points, the minimal important change was 17.2 points, and there were no floor or ceiling effects for the score.

## Introduction

Patellofemoral pain is usually located around the kneecap and increases with squats and climbing stairs, and among other activities, such as running [1, 2]. It affects one in every four athletes and 2.5 million college students every year in the USA [3, 4]. It is the most common cause of consultation in sports medicine clinics and represents 25% of knee problems [4, 5]. Patellofemoral pain may be part of a degenerative joint disorder in middle-aged and older populations with patellofemoral osteoarthritis.

There is a lack of objective disease-specific scores for patellofemoral pain and osteoarthritis. The Kujala score was the first score developed for patellofemoral pain and has been translated to several languages, including Spanish. However, there have been criticisms of it regarding some technical and difficult-to-understand questions. Thus, it is desirable to have additional scores to evaluate patients with patellofemoral pain. In 2018, Crossley et al. published and validated the patellofemoral pain and osteoarthritis subscale KOOS-PF [6], a self-administered score that measures pain, stiffness and quality of life in relation to patellofemoral pain and osteoarthritis. The KOOS-PF is a useful tool for evaluating patients in clinical practice and for measuring outcomes.

Only one translation of the KOOS-PF score has been published at this point (Arabic), but there are several studies currently on-going. This study aimed to translate to Spanish and validate the Spanish version of the KOOS-PF score.

## Main text

### Materials and methods

This was a validation study for the Spanish version of the KOOS-PF score following the COSMIN (Consensus-based Standards for the selection of health Measurement Instruments) guidelines [7]. It was conducted in two parts: (i) Subscale translation to Spanish and (ii) Evaluation of measurement properties. Patients with the diagnosis of interest were invited by their orthopaedic surgeon from their clinic at Fundación Valle del Lili in Cali, Colombia. The Biomedical Research Ethical Committee from the hospital approved the study. Consent form was waived by the IRB because these scores are part of the usual assessment for these patients.

#### Translation and cross-cultural adaptation

The authors of the subscale were contacted, and permission was obtained to translate the KOOS-PF to Spanish. The original version includes 11 self-assessment questions grouped into three different categories: stiffness, pain and quality of life. These questions have five possible answers, represented by a box that the participant should tick. Each answer has a numeric value from 0 to 4. The final score is on a 0–100 scale, where 0 is the worst and 100 the best health score.

The translation followed the recommendations by Beaton et al. [8]. Two independent forward translations were performed from the original English version by an orthopedic surgeon (JPM-C) fluent in English whose native language was Spanish and a professional language translator. The two versions were then conciliated. This version was then back-translated by two native English-speaking persons, a medical doctor and a professional language translator. They were not familiar with the original English version of KOOS-PF. A final conciliation between the translators with both versions was performed to obtain a Spanish version that was tested in a pilot group of five patients with patellofemoral pain syndrome. This test allowed us to evaluate the understanding of the questions by the patients and to identify any problems in answering the items. The Spanish version of the KOOS-PF is available free of charge from http://www.koos.nu.

#### Evaluation of measurement properties

The measurement properties included reliability (internal consistency, test–retest reliability and measurement error), responsiveness and interpretability (smallest detectable change (SDC), minimal important change (MIC), minimal important difference (MID) and floor and ceiling effects). Sample size was calculated to be 50 patients, with 90% power to detect intraclass correlation coefficient as low as 0.4 in two different tests. Stata 14.0 was the software used.

#### Internal consistency

Using baseline KOOS-PF data, we calculated Cronbach’s alpha, with values between 0.7 and 0.95 considered to be adequate [9]. A lower value suggests poor correlation among subscale items and limits the interpretability of the total overall score. A very high value suggests item redundancy.

#### Test-rest reliability

The intraclass correlation coefficient (ICC) was used to evaluate reliability. For this calculation, patients were asked to answer the score again 7 days after the baseline measurement, and the reliability between those two tests was calculated. Values ≥ 0.7 were considered adequate [9]. We also calculated the standard error of the mean (SEM), which is equivalent to SDx√1 − ICC (where SD is the standard deviation of the observed scores) [10]. Finally, we used a Bland–Altman plot to confirm homoscedasticity [11].

#### Responsiveness

To evaluate responsiveness, we used the global rating of change (GROC) score, which is a single-item questionnaire where the patient had five possible options to answer according to their change in knee pain one month after the baseline measurement. The five response options (Likert scale) ranged from ‘much worse’ (score of 0), ‘slightly worse’ (score of 1), ‘about the same’ (score of 2), ‘slightly better’ (score of 3), to ‘much better’ (score of 4). All patients were treated with physical therapy during this month to evaluate responsiveness to this type of treatment. Not all patients were adherent to therapy.

#### Convergent validity

We evaluated the correlation between the KOOS-PF and the Spanish version of the Kujala score [12]. The Kujala score has 13 items relating to symptoms and activities associated with anterior knee pain. Each item has between 3 and 5 possible answers, with some items scoring between 0 5 points and others scoring 0–10 points. The final score is in the range of 0–100, where 100 represents perfect health. We used Pearson’s correlation coefficients to evaluate convergent validity.

#### Interpretability

We defined floor and ceiling effects as 15% or more of the sample scoring the lowest or highest possible score on the KOOS-PF. The smallest detectable change was estimated at 90% confidence interval (SDC90), for individual changes as 1.65x√2xSEM, and for group changes as 1.65x√2xSEM/√n. [13].

The minimal important change (MIC) was estimated using the mean KOOS-PF change in score between the baseline and 1-month measurements, for patients reporting to be ‘slightly better’ 1 month later, using the GROC (score of 3). Meanwhile, the minimal important difference (MID) was estimated as the difference in mean change scores between patients reporting being ‘slightly better’ and those feeling ‘about the same’ (score of 2) [13].

### Results

#### Study participants

Between June 2019 and March 2020, sixty patients with patellofemoral pain (73%)/osteoarthritis (27%) completed the Spanish version of the KOOS-PF at baseline, 58 patients seven-days later for the test–retest evaluation and 55 patients one-month after for the responsiveness and meaningful changes. Table 1 shows the baseline characteristics of the participants. The mean age was 31 years old (range: 12–64). Most were women (75%) and had bilateral pain (41%). The mean score (SD) for the KOOS-PF at baseline was 46 (23), and at the 30-day follow-up, it was 61 (24).

**Table 1:** Baseline characteristics of the study participants with patellofemoral pain in this study

Variables	n = 60
Age, mean ± SD	30.95 ± 10.7
Sex, n (%)	
Female	45 (75)
Male	15 (25)
Knee, n (%)	
Right	17 (28)
Left	16 (27)
Both	27 (45)

#### Internal consistency

Internal consistency was very high (Cronbach’s alpha = 0.93).

#### Test–retest reliability

**Fig. 1:**
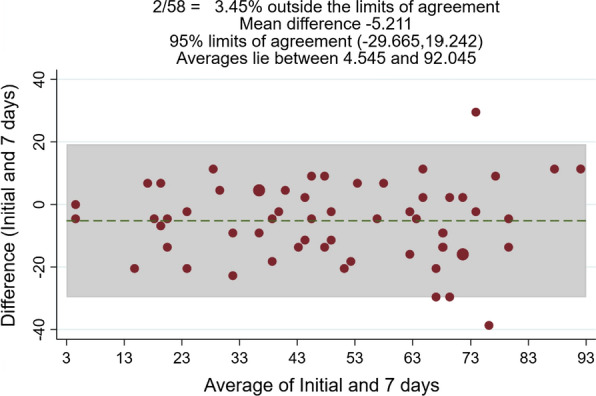
Bland–Altman plot for the agreement between test–retest measurements of the KOOS-PF

For participants who completed both the baseline and retest questionnaires within 1-week (n = 58), the score demonstrated very high test–retest reliability, with an ICC of 0.82 (95% CI 0.697–0.902) and an SEM of 3.7. The Bland–Altman plot showed no systematic differences between the first and second measurements for the KOOS-PF score (Fig. 1).

#### Responsiveness

**Fig. 2:**
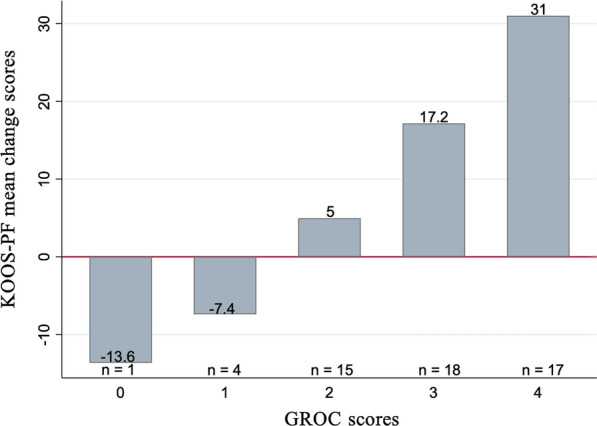
KOOS-PF mean change scores (negative change represents worsening) vs GROC scores (0 = much worse; 1 = slightly worse; 2 = no change; 3 = slightly better; 4 = much better) demonstrated good correlation (r = 0.64)

The KOOS-PF change scores showed a good correlation with GROC scores (r = 0.64) (Fig. 2).

#### Convergent validity

The KOOS-PF showed a strong positive correlation with the Spanish version of the Kujala score (r = 0.71). The scatter plot for this correlation.

#### Interpretability

There were no floor or ceiling effects for the KOOS-PF with no patients with minimum/maximum scores. The individual SDC90 was 8.6, and for the group, the SDC90 was 1.1. The MIC was 17.2, and the MID was 11.1.

### Discussion

This study shows the process for translation and validation of the Spanish version of the KOOS-PF subscale. This questionnaire showed strong psychometric properties, strong correlation with similar scores, such as the Kujala score, and a good responsiveness correlation with the GROC score. These findings are very similar to what was shown with the original English version of the KOOS-PF subscale [6].

Additionally, similar results were found in the validation of the Arabic version. While our internal consistency was very good, with a Cronbach’s alpha of 0.93, Ateef [14] reported a Cronbach’s alpha of 0.81. However, regarding reliability, the Arabic translation had an ICC of 0.96 [14], which was better than our ICC of 0.82. This difference in the intraclass correlation coefficient could be explained by the timing between the first and second questionnaires administration, with intervals of 48-h in the former study and 7-days in the latter one. Similarly, none of the translations found floor or ceiling effects.

This study follows the COSMIN checklist for cross-cultural validation [7], showing that the Spanish questionnaire is a reliable, valid and responsive measurement tool for use in patients with patellofemoral pain and osteoarthritis. In terms of the smallest detectable change, the score was able to detect very small changes for the whole group (1.1), with still a low number for the individual-level changes (8.6). These values are even smaller than for the original score [6].

The KOOS-PF showed minimal important differences and minimal important change values that were very similar to the English score values. These data are important for the interpretation of future studies regarding the evaluation of treatments for patellofemoral pain or osteoarthritis. It is crucial for clinicians to understand how much difference or change is clinically important for patients.

This is the second validation for a translation of the KOOS-PF subscale. This type of translation and validation permits clinicians and researchers to develop studies using a valid score.

### Conclusions

The Spanish version of the KOOS-PF demonstrated very good measurement properties, including internal consistency, reliability and responsiveness. The KOOS-PF can be used in Spanish-speaking patients for clinical and research purposes in patellofemoral pain and osteoarthritis.

## Limitations

The second questionnaire was administered within one week of the baseline questionnaire, during which time some participants could start physical therapy, and some of their symptoms would improve. However, Crossley et al. [6] administered the second questionnaire within two weeks of the first questionnaire. Generally, it is considered that one week is enough time to prevent recall and not enough time to see improvements. In addition, our very good ICC shows that this difference did not affect the results in a considerable way.

## Data Availability

The datasets used and/or analysed during the current study are available from the corresponding author on reasonable request. The Spanish version of the KOOS-PF is available free of charge upon request to the corresponding author.

## Abbreviations

KOOS-PF: Patellofemoral pain and osteoarthritis subscale of the knee injury and osteoarthritis outcome score
GROC: Global rating of change
PROM: Patient-reported outcome measure
COSMIN: Consensus-based Standards for the selection of health Measurement Instruments
ICC: Intraclass correlation coefficient
SDC: Smallest detectable change
MIC: Minimal important change
MID: Minimal important difference
SEM: Standard error of the mean
SD: Standard deviation
